# Levothyroxine Prescription Error: A Case Report

**DOI:** 10.7759/cureus.44787

**Published:** 2023-09-06

**Authors:** Michael J Valentine, Hunter D Kramer, James Kim, Nicholas J Pettinelli, Robert J Beers

**Affiliations:** 1 Family Medicine, Kansas City University, Kansas City, USA; 2 Family Medicine, Utah Carenow, South Jordan, USA

**Keywords:** thyrotoxicosis, iatrogenic, error, levothyroxine, prescription error, dosing error, synthroid, suicidal ideation, levothyroxine overdose, levothyroxine poisoning

## Abstract

Levothyroxine (LT) is the synthetic form of thyroxine (T4), a thyroid hormone analog used to treat hypothyroidism. LT overdose rarely results in severely poor outcomes. General guidelines for treating exogenous thyrotoxicosis depend on the severity of symptoms. There is no standardized protocol; however, drug discontinuation, beta-blockers (specifically propranolol), and cholestyramine effectively manage overdose when needed, with most cases resolving independently without medical intervention.

Here, we present the case of a 26-year-old female with a history of supraventricular tachycardia, anxiety, depression, and Hashimoto thyroiditis who was accidentally overprescribed LT (300 mcg for one and a half months) that resulted in symptoms of lethargy, tremors, body temperature dysregulation, orthostatic hypotension, and diarrhea. This case, with limited evidence, suggests that excessive LT exacerbated the patient's underlying psychiatric symptoms, encouraging suicidal ideation.

## Introduction

In the United States, hypothyroidism prevalence is approximately 11.7% of the population with more than 78% of cases receiving thyroxine (T4) monotherapy [1]. Hypothyroidism is typically managed with lifelong thyroid hormone supplementation with levothyroxine (LT), a synthetic form of T4. The Food and Drug Administration (FDA) has placed a black box warning on LT, indicating that it should not be used for weight loss to treat obesity. Controversially, this medication has been shown to increase metabolism, leading some patients to misuse the substance with intent. Notwithstanding, factitious thyrotoxicosis is more likely to be seen in the setting of accidental ingestion or in the form of supplemental diet pills [2,3]. Furthermore, there have been reports of suicidal attempts by LT ingestion [4].

A study has postulated that 10-fold medication dose errors are commonplace throughout health care. Out of 200 consecutively documented dosage errors, they found that overdose cases were accidentally prescribed in 61% (122/200) of order errors. Interestingly, 19% (38/200) of the errors involved LT [5]. 

## Case presentation

A 26-year-old female with a significant history of supraventricular tachycardia, depression, and generalized anxiety disorder (GAD) presented to an outpatient clinic for a second opinion. Six months ago, the patient presented to her therapist after having a two-day history of suicidal ideation. The patient was then referred to a psychiatrist. The psychiatrist performed an interview in which the patient complained of pounding heartbeats, temperature dysregulation, anxiety, and depression that had progressively worsened for the past month and a half and then culminated into suicidal ideation. It is worth noting that the patient had experienced suicidal ideation over a decade ago, but no recent incidents had been reported until the recent episode. The patient was also taking bupropion (100 mg, two times a day) and escitalopram (10 mg, every day).

Upon review of medications, the psychiatrist found the patient's LT prescription dosage to be abnormally high at 300 mcg. Such a dosage is rarely prescribed and is typically reserved for patients with poor compliance, drug interactions, or malabsorption. The psychiatrist then called the patient’s primary care provider (PCP), and the mistake was confirmed in the PCP’s electronic record system. The PCP had switched the patient to a higher dose from 25 mcg to 50 mcg for Hashimoto thyroiditis management. However, due to a mistake, the clinic had accidentally sent a prescription order to the pharmacy for 300 mcg instead of 50 mcg. After a discussion, the psychiatrist and the PCP concluded that a trip to the emergency room was unnecessary. The patient would be managed clinically as an outpatient. They recommended that the patient discontinue her LT and would follow up with her via phone calls and messages. 

It is also worth noting that in our encounter, the patient presented all of the documentation she had from her PCP. Unfortunately, there was no past record of the patient's thyroid-stimulating hormone (TSH) level at the time of her clinical manifestations. However, there were records of her TSH and free T4 levels before the prescription error and about two months after the suicidal ideation event. These levels were 1.87 uIU/mL and 3.86 uIU/mL, respectively (reference range of 0.45-5.33). Her free T4 levels were 1.01 ng/dL and 0.79 ng/dL, respectively (reference range of 0.59-1.64). Her blood work was also normal. Notably, it is concerning that additional blood work was not ordered at the time of the incident.

It is possible that the PCP knew the patient was in iatrogenic LT thyrotoxicosis but decided to diagnose and manage it clinically. Two months after the thyrotoxicosis incident, the PCP advised the patient to continue taking 25 mcg of LT and to report any abnormal symptoms. During another follow-up with the PCP, the patient reported that her anxiety and depression had remained, albeit not as amplified as before. A PHQ-9 (patient health questionnaire) and GAD test were administered by her PCP and showed a worsening condition when compared to previous questionnaires despite previous attempts at management. Notably, the PCP did not record the questionnaire scores. Her PCP then diagnosed the patient with treatment-resistant depression, and the patient was treated with multiple sessions of transcranial magnetic stimulation (TMS). 

In our encounter, the patient was reassured, educated about medical errors, and then scheduled for follow-up. Objectively, the patient did not appear to be in any distress and was well groomed. Her reported mood was euthymic, but her affect seemed mildly dysphoric. In addition, she did get flustered when recounting the incident. After a month, the patient reported that 80-90% of her psychiatric symptoms had disappeared after the completion of TMS therapy.

## Discussion

LT has a relatively long half-life (7.5 days), but LT poisoning rarely results in a severe outcome. A study by Nygaard et al. reported that acute LT toxicity is typically benign, followed by late onset of symptoms. However, they ultimately concluded that all symptoms resolved without needing medical care and that symptoms did not appear to be dose-dependent [6]. 

Common signs of LT toxicity may include anxiety, tremors, tachycardia, flushing, and diarrhea [3]. Additionally, interference with normal homeostatic functions may occur [7]. Ribeiro et al. describe the dependence of the sympathetic nervous system on thyroid hormone in the production of shivering when rats are cold [8]. In this case, the excessive amounts of LT in the patients’ system resulted in the clinical response of temperature dysregulation, underscoring a distinguishing physical examination finding that highly suggested the diagnosis.

The management of LT overdose symptoms is similar to thyrotoxicosis management [3]. A few medications have beneficial effects, with propranolol as the preferred choice. This is because propranolol (a beta-blocker) inhibits the peripheral conversion of T4 to T3 and is a non-selective beta-adrenoreceptor antagonist. Other potential medications may include activated charcoal and cholestyramine. In particular, cholestyramine will decrease thyroxine absorption by binding to T4 and increase drug clearance. Bateman et al. encourage therapeutic titration in response to LT toxicity symptoms with the recommendation of exogenous thyroid hormone discontinuation. In severe cases of thyroid storm induction, extracorporeal removal with intensive care unit admittance may be considered [3,9]. 

Some types of medications have been reported to contribute to suicidal ideation. These commonly include antidepressants and antiepileptics. It is imperative to point out that the relationship between medications and suicidal ideation is not fully understood. This remains to be highly controversial and is debated rigorously [10,11]. Often, confounding variables such as underlying mental health conditions and family history play a role in combination with medication to result in suicidal ideation, therefore making the association of medication with suicidal ideation not readily apparent.

Given the infrequency of iatrogenic LT toxicity, it is difficult to establish a direct relationship between LT toxicity and suicidal ideation. Neuropsychiatric side effects of LT toxicity may include symptoms like anxiety, agitation, nervousness, and insomnia [12]. Interestingly, a systematic review and meta-analysis found no substantial association between thyroid dysfunction and suicidal behavior [13]. A thorough study design with strict controls for confounding variables would be crucial to draw a definitive correlation. Given the constraints of our encounter, we can only postulate that increased anxiety and depression may have contributed to this patient’s predisposed suicidal tendencies as an adverse effect from LT.

## Conclusions

Prescription errors are commonplace in the healthcare field. Reported iatrogenic LT toxicity is rare. Overdose symptoms include anxiety, agitation, nervousness, palpitations, and tremors. In addition to these findings, our patient presented with depression exacerbation that culminated into suicidal ideation. With this report, we hope to highlight the importance of regular surveillance of our patients after starting new medications, with emphasis on a thorough review of systems and review of medications.

In conclusion, acute LT overdose is commonly benign, with long-term clinical responses of symptoms that will resolve independently. Management can be effectively done with propranolol and cholestyramine but, in most cases, does not require medical intervention. It is possible that neuropsychiatric manifestations as an adverse effect from iatrogenic LT overprescription were enough to exacerbate this patient's underlying psychiatric symptoms, particularly depression and anxiety, to ultimately encourage suicidal ideation. However, there is limited evidence. We hope that this unique case reminds physicians to double-check prescription orders, to consider review of medications in their patient encounters, and above all else, to do no harm.
